# Is Salvage Hyperbaric Oxygen Therapy Effective for Sudden Sensorineural Hearing Loss in Patients with Non-response to Corticostreoid Treatment?

**DOI:** 10.7759/cureus.6560

**Published:** 2020-01-04

**Authors:** Ayşe Seçil Kayalı Dinç, Melih Çayönü, Süleyman Boynueğri, Evrim Ünsal Tuna, Adil Eryılmaz

**Affiliations:** 1 Otolaryngology, Ankara Bilkent City Hospital, Ankara, TUR

**Keywords:** sudden sensorineural hearing loss, hyperbaric oxygen therapy, corticosteroid, salvage treatment

## Abstract

Purpose: The aim of this study was to evaluate the efficacy of salvage hyperbaric oxygen therapy (HBOT) for sudden sensorineural hearing loss (SSNHL); HBOT is performed after three weeks of the onset of the disease.

Methods: This retrospective clinical study included patients with unilateral idiopathic SSNHL. All patients admitted to the hospital with the diagnosis of SSNHL were given standard steroid treatment within the 14 days of the onset of the SSNHL. We compared the two study groups - Group A: patients receiving steroid treatment within the first 14 days; Group B: patients receiving corticosteroid treatment within the first 14 days, but unresponsive to this treatment, and began to receive HBOT after three weeks of the onset of SSNHL for the purpose of salvage therapy.

Results: A total of 50 patients were included in the study. The mean age of the patients was 50.6 ± 14.1 years. There was not a significant difference in the degree of hearing loss between the groups based on the findings from audiometric examinations performed at the time of diagnosis. It was observed that salvage HBOT was not effective when the initial and post-treatment audiometric tests were compared.

Conclusion: According to our results, salvage HBOT was not efficient when performed three weeks after the onset of the SSNHL for patients who did not respond to corticosteroid treatment.

## Introduction

Sudden sensorineural hearing loss (SSNHL) is one of the most important clinical emergencies in the otologic era with a chance of recovery with treatment. It is characterized by hearing loss of at least 30 dB in three consecutive sound frequencies in audiometric tests, which develops in 72 hours [1]. Although the incidence of SSNHL has been reported to range from 5 to 20 per 100,000, it is recognized that this rate is higher actually [2-3]. The severity of hearing loss occurs on a scale ranging from moderate to total loss, which may lead to deafness and social distress in the patients [3].

No consensus has been reached on the proposed treatment protocols of this frequent disease. In patients with known etiology, the treatment is directed to the underlying cause. However, in the majority of the cases, the etiology remains unknown and corticosteroid therapy is given to all of the patients as it is the only mode of treatment with proven efficacy currently [4-5].

The main rationale of hyperbaric oxygen therapy (HBOT) is the prevention of ischemia and the recovery from hypoxia to restore cell functions by administering high-pressure oxygen. It is suggested that ischemia due to vascular damage is involved in developing SSNHL. Thereby, it is assumed that ischemia-induced impaired hearing will be improved with HBOT by increasing the availability of oxygen in the inner ear [6-7]. Although the HBOT for SSNHL is suggested within two weeks of the onset of the disease, HBOT can also be suggested as salvage therapy within one month of onset of SSNHL [5].

Different results in favor and against are available in the literature in terms of salvage HBOT for SSNHL [8-10]. Therefore, the aim of this study was to evaluate the efficacy of salvage HBOT for SSNHL, which is performed after three weeks of the onset of the disease. For this purpose, we compared the two study groups: Group A: patients receiving steroid treatment within the first 14 days; Group B: patients receiving steroid treatment within the first 14 days, but unresponsive to treatment, and began to receive HBOT after three weeks of the onset of the SSNHL for the purpose of salvage therapy.

## Materials and methods

This retrospective clinical study included patients with unilateral idiopathic SSNHL who were hospitalized and treated in the otorhinolaryngology clinic of Ankara Numune Training and Research Hospital during the period from December 2013 to March 2019. All investigations were performed in accordance with the declaration of Helsinki on biomedical studies involving human subjects, and informed consent was obtained from all study subjects.

Demographic data of the patients (identity information, age, gender), side of SSNHL (right or left ear), laboratory test results (hemogram, hemostasis, and biochemical parameters), audiometry results (before and after treatment), magnetic resonance imaging results and the treatment protocols were retrieved from the hospital automation system and recorded in Statistical Package for the Social Sciences; version 21.0 (SPSS Inc., Chicago, IL) software.

The patients were excluded when SSNHL developed due to varicella-zoster infections, cerebellopontin tumors, or other cranial pathologies, when SSNHL occurred in both ears, when patients had a previous history of hearing loss, and when patients had a history of chronic otitis media or ear surgery.

All patients admitted to the hospital with the diagnosis of SSNHL were given standard steroid treatment within the 14 days of the onset of the SSNHL. HBOT was recommended to all patients. However, some patients received HBOT after three weeks of the onset of SSNHL because they preferred to wait for the outcome of the steroid treatment for individual reasons. Thus, those patients who received salvage HBOT were also non-responsive to steroid treatment.

Methylprednisolone (1 mg/kg) was started in patients without contraindications for steroids. It was discontinued by reducing 10 mg every two days. All the patients also received pirecetam 800 mg, three times a day with pantoprazole 40 mg once a day.

HBOT was administered in a single session at 2.4 ATA pressure for 20 days. Patients were ventilated with 100% pure oxygen at each 120-minute session and in three periods per 20-minute session. There was a 20-minute break between the periods.

Two study groups were constituted for our study. Group A included patients receiving steroid treatment within the first 14 days, whereas Group B included patients receiving steroid treatment within the first 14 days and began to receive HBOT after three weeks of the onset of SSNHL. The audiometric tests which were performed at the initial and at the end of the second month of treatment were used for the comparisons between the study groups.

## Results

A total of 50 patients, meeting the inclusion criteria, were included in the study. Of these, 15 (30%) were females and 35 (70%) were males. The age range of the patients was from 19 to 79 years. The mean age of the patients was 50.6 ± 14.1 years (mean ± SD).

The number of patients who received only medical treatment (Group A) was 28 (56%) and the number of patients receiving salvage HBOT (Group B) was 22 (44%). There were no significant differences in gender distribution, age, and laboratory results between the groups. Also, there was not a significant difference in the degree of hearing loss between the groups based on the findings from audiometric examinations performed at the time of diagnosis (Table 1). As shown in Table 1, Group A had a significant improvement in post-treatment audiometric tests when compared to Group B. Moreover, it was shown that salvage HBOT was not effective when the initial and post-treatment audiometric tests were compared (Table 2).

**Table 1:** The descriptive statistics of the study groups (p < 0.05 was accepted as statistically significant)

	Only medical treatment (Group A)	Salvage HBOT after medical treatment (Group B)	P value
Age	53.1 ± 11.5	47.5 ± 16.8	0.17^+^
Gender (female/male)	7/21	8/14	0.38^*^
Initial bone hearing levels	52.4 ± 12.3	57.2 ± 14.7	0.21^+^
Post-treatment bone hearing levels	32.9 ± 24	66.1 ± 32.3	< 0.001^+^

**Table 2:** HBOT treatment results (p < 0.05 was accepted as statistically significant) HBOT: hyperbaric oxygen therapy.

	Initial bone hearing levels	Bone hearing levels after salvage HBOT	P value
Salvage HBOT after medical treatment (Group B)	57.2 ± 14.7	66.1 ± 32.3	0.13^*^

## Discussion

Our results showed one major finding - salvage HBOT performed after three weeks of SSNHL onset was not effective in the treatment of SSNHL in patients with non-response to the corticosteroid treatment.

Etiology and treatment of SSNHL have been a subject of continuous debate for years. The incidence of this disease is 16 per thousand and approximately 32%-70% of the people recover spontaneously without consulting a doctor [3-4]. Although spontaneous recovery rates seem to be possible, persistent hearing losses of moderate to severe degrees are also possible in SSNHL. Therefore, treatment should be started immediately after the diagnosis [5].

More than 60 variations of medication combinations have been tried for the treatment of SSNHL so far. Medications or treatment modalities used for the treatment include oral steroids, vasodilators, vitamins, antioxidants, antivirals, and HBOT [11]. However, the most common active agent used currently in the treatment is corticosteroids undoubtedly [5]. Standard steroid therapy is the first-line treatment option in SSNHL [5]. The rationale for using corticosteroids for the treatment of SSNHL is based on the hypothesis suggesting that steroids can prevent or reverse the inflammation in the internal ear [12].

The starting time of the treatment after the onset of the SSNHL was an important issue which had a direct effect on the improvement of hearing loss. Treatment with corticosteroids appeared to be best for the recovery in the first two weeks, with little benefit after four to six weeks. Also, it was found that the recovery was mostly detected during the first two weeks of the corticosteroid treatment, whereas, late recovery has also been reported [5,13]. In a similar fashion, timing might be also important for HBOT in the treatment of SSNHL. HBOT can be used as a primary treatment modality combined with corticosteroids or as a salvage therapy for patients with non-response to corticosteroids. A recent guideline recommended the use of corticosteroid treatment plus HBOT within two weeks after the onset of the SSNHL [5]. Also, it was found that the combination of oral steroid therapy and HBOT was the most effective when compared to oral steroid therapy, intratympanic steroid therapy, and HBOT alone [14]. Moreover, in another study, researchers found that hearing gain was significantly low when HBOT was performed after 14 days of SSNHL [10]. On the contrary, HBOT can be performed until one month of the diagnosis in terms of salvage therapy [5]. In our study, HBOT was given to 22 patients, who had failed to recover with corticosteroid treatment. HBOT was started on the third week of the onset of SSNHL, however only two patients had partial recovery, and the majority of the salvage HBOT group showed no improvement in terms of hearing levels.

There is still too much unknown information about this disease. It is so regrettable that we have no idea as to which patient will recover and which will not, despite the all the known prognostic factors. As known, spontaneous recovery is possible in this disease, however, we do not know for whom we have applied over-treatment. That is why further investigations are needed to be done on this subject.

The major limitation of our study was its retrospective design and not allowing for the evaluation of the efficacy of individual treatment protocols. Also, another limitation of our study was the low number of study patients.

## Conclusions

Satisfactory results can not be achieved in salvage HBOT, performed after three weeks from the onset of SSNHL in patients with non-response to corticosteroid treatment.
